# Synthetic Musk Compounds: Luckenbach Responds

**Published:** 2005-12

**Authors:** Till Luckenbach, David Epel

**Affiliations:** Hopkins Marine Station of Stanford University Pacific Grove, California E-mail: depel@stanford.edu

In his letter on our recent article in *EHP* (Luckenbach and Epel 2005), Salvito raises important questions about effects of the synthetic musk fragrances regarding *a*) human and environmental health effects, *b*) environmental concentrations of the musks, and *c*) uniqueness of inhibition of efflux transporters to the musks, the effect we described in our article.

*a*) Regarding health issues, we agree with Salvito that the available evidence indicates minimal direct affects of most synthetic musks on the health of humans and aquatic organisms. However, our data expand the definition of toxicity and detrimental effects to indirect and unanticipated consequences of these chemicals, even if the chemical itself might be nontoxic. The major point of our article (Luckenbach and Epel 2005) was that the musks inhibit efflux (drug) transporters, which act as first lines of defense to pump potentially toxic substances out of cells. These efflux transporters are ubiquitous and are found in bacteria, fungi, plants, and animals, including humans. The transporters have wide substrate specificity, and this binding to many compounds can result in inhibition of activity by competing substrates. As a consequence of transporter inhibition, cells and organisms can therefore become exposed to toxicants normally kept out of their cells.

An unexpected finding was not only that the musks inhibit these transporters in marine mussels but that the effect is long-term and persists up to 24–48 hr after removal of the musk compounds. These indirect and long-term toxicity effects are of particular concern because these chemicals are stable and bioaccumulate; for example, musk xylene has a half-life of 70 days in human tissue (Riedel and Dekant 1999).

Effects on human transporters by the musks cannot be inferred from our results, but they do point to the possibility of an interaction, considering the general property of the transporters to recognize a wide array of substrates. Therefore—and in light of accumulation of the musks in human tissue—research is needed to determine if the musks similarly inhibit the human efflux transporters, thereby compromising this defense against toxicants.

*b*) The musks are of environmental concern because they enter the water column from incomplete degradation in sewage plants. We agree with Salvito that the reported levels in surface waters are extremely low (picomolar range) but disagree with his conclusion that such levels indicate that musks are not a problem. In spite of these low environmental levels, there is significant bioaccumulation of these chemicals in tissues of mussels and fish, and just several months ago Nakata (2005) reported significant bioaccumulation in cetaceans. The concentrations in aquatic organisms can become quite high, being on the order of nanograms per gram fresh weight, which translates to about 0.1 μM final concentration in tissue (Nakata 2005; Rimkus 1999; Yamagishi et al. 1983).

According to Salvito, worldwide production of synthetic musks are only about one-half of the amount we cite. These lower numbers are even more worrisome because because this means that the potency of the musks to bioaccumulate is even higher.

*c*) Salvito points out that the inhibition of transporters is not unique to the musks. We agree and note that the observed inhibition of efflux transporter activity by the musks may be the tip of the iceberg. As with the musks, there may be many chemicals that by themselves are not toxic but similarly inhibit the efflux transporters and thereby expose the organism to normally excluded toxicants.

In summary, the available data suggest that efflux transporter inhibition could be a significant indirect, negative, and unappreciated effect of environmental chemicals. Several questions need to be answered: Do these chemicals inhibit human transporters? Are there other anthropogenic and natural products that inhibit these transporters in aquatic organisms and also in humans? Should anthropogenic chemicals be screened for inhibitory activity? If so, should there be voluntary or governmental regulations to ensure that such chemicals do not affect the health of exposed populations through these indirect actions?
